# Introducing the Futile Recanalization Prediction Score (FRPS): A Novel Approach to Predict and Mitigate Ineffective Recanalization after Endovascular Treatment of Acute Ischemic Stroke

**DOI:** 10.3390/neurolint16030045

**Published:** 2024-05-30

**Authors:** Helen Shen, Bella B. Huasen, Murray C. Killingsworth, Sonu M. M. Bhaskar

**Affiliations:** 1 Global Health Neurology Lab, Sydney, NSW 2150, Australia; 2 South West Sydney Clinical Campuses, UNSW Medicine and Health, University of New South Wales (UNSW), Sydney, NSW 2170, Australia; 3 Department of Interventional Neuroradiology, Lancashire Teaching Hospitals NHS Foundation Trust, Preston PR2 9HT, UK; 4 Edinburgh Medical School, College of Medicine and Veterinary Medicine, University of Edinburgh, Edinburgh EH16 4UX, UK; 5 Ingham Institute for Applied Medical Research, Cell-Based Disease Intervention Group, Clinical Sciences Stream, Liverpool, NSW 2170, Australia; 6 NSW Brain Clot Bank, NSW Health Pathology, Sydney, NSW 2170, Australia; 7 Department of Anatomical Pathology, NSW Health Pathology, Correlative Microscopy Facility, Ingham Institute for Applied Medical Research and Western Sydney University, Liverpool, NSW 2170, Australia; 8 Department of Neurology & Neurophysiology, Liverpool Hospital, South West Sydney Local Health District, Liverpool, NSW 2170, Australia

**Keywords:** acute stroke, endovascular thrombectomy, futile recanalization, prognosis, clinical score, risk prediction

## Abstract

*Objective*: This study aims to develop and validate the Futile Recanalization Prediction Score (FRPS), a novel tool designed to predict the severity risk of FR and aid in pre- and post-EVT risk assessments. *Methods*: The FRPS was developed using a rigorous process involving the selection of predictor variables based on clinical relevance and potential impact. Initial equations were derived from previous meta-analyses and refined using various statistical techniques. We employed machine learning algorithms, specifically random forest regression, to capture nonlinear relationships and enhance model performance. Cross-validation with five folds was used to assess generalizability and model fit. *Results:* The final FRPS model included variables such as age, sex, atrial fibrillation (AF), hypertension (HTN), diabetes mellitus (DM), hyperlipidemia, cognitive impairment, pre-stroke modified Rankin Scale (mRS), systolic blood pressure (SBP), onset-to-puncture time, sICH, and NIHSS score. The random forest model achieved a mean R-squared value of approximately 0.992. Severity ranges for FRPS scores were defined as mild (FRPS < 66), moderate (FRPS 66–80), and severe (FRPS > 80). *Conclusions*: The FRPS provides valuable insights for treatment planning and patient management by predicting the severity risk of FR. This tool may improve the identification of candidates most likely to benefit from EVT and enhance prognostic accuracy post-EVT. Further clinical validation in diverse settings is warranted to assess its effectiveness and reliability.

## 1. Introduction

Acute ischemic stroke (AIS) poses an enormous burden across the world and ranks as the second leading cause of death [1]. The 2019 Global Burden of Disease (GBD) study indicated that there were 12.2 million stroke incidents, resulting in 143 disability-adjusted life years (DALYs) and 6.55 million deaths, with projections indicating an increase in these numbers [2]. In the era of reperfusion therapy, intravenous thrombolysis (IVT) and endovascular thrombectomy (EVT) are currently the mainstays of AIS treatment, recommended by the American Heart Association (AHA) and the American Stroke Association (ASA) [3]. However, the benefits are inconsistent across all AIS patients, with a small subgroup failing to derive therapeutic benefits [4]. An emerging area of clinical and research interest is futile recanalization (FR), defined as successful recanalization without therapeutic benefits [4]. However, the exact mechanism behind FR remains partially understood, and discrepancies persist across studies regarding its prevalence, predictors, and outcomes [5].

Understanding the risk and occurrence of FR and identifying associated factors is crucial for physicians to distinguish patients likely to benefit from EVT. This knowledge can reduce the number of futile interventions, elucidate underlying prognostic factors, and facilitate comprehensive management planning for patients undergoing EVT. A risk prediction score to mitigate harm among subgroups of patients at risk of FR is warranted. However, minimal studies have explored this area [6].

In this study, we introduce the Futile Recanalization Prediction Score (FRPS), a novel tool designed to predict the severity risk of FR after EVT. We provide details on its methodological development and discuss various case scenarios for its application in clinical studies. The FRPS aims to enhance treatment planning and patient management by offering a reliable means to predict FR risk, thereby improving outcomes for AIS patients.

## 2. Methodology

Supplemental Information S1 provides a detailed overview of the definitions, predictors, outcomes after FR, and various treatment considerations relevant to FR. Table 1 summarizes key findings published in the meta-analyses on the reported prevalence, predictors of FR, and association with outcomes. Based on the indicators associated with FR in previous literature [7,8,9] and analyzed here, we propose a novel score, the FRPS, which incorporates multiple factors to predict the risk severity of FR after EVT.

### Development, Optimization, and Modeling Simulation for Risk Prediction Score for Predicting Futile Recanalization Risk Severity after Endovascular Thrombectomy

The FRPS was developed using a rigorous process as follows:

We selected predictor variables based on their clinical relevance and potential impact on the FRPS score. All mathematical modeling and simulation, including statistical analyses, were performed using Python. The initial equation for the FRPS was derived from Shen et al.’s meta-analysis [7]. Additionally, we explored other models incorporating clinically relevant variables. To determine the most effective model, we conducted various analyses, including calculating mean scores, plotting histograms of FRPS scores, evaluating statistical measures like R-squared, and performing cross-validation to assess generalizability.

Our initial modeling revealed negative R-squared values, suggesting linear regression might not be ideal. We explored nonlinear regression techniques and selected predictor variables with stronger linear relationships. Ultimately, we constructed a new model using a subset of predictor variables based on clinical relevance and statistical significance (Supplemental Information S2 Online for Python Code). We employed a machine learning algorithm (a random forest regression model) using the selected variables, which effectively captures nonlinear relationships. We also evaluated the model’s performance using cross-validation with five folds and computed the R-squared value across all folds to assess the model’s fit to the data.

The final equation for the FRPS model is

$$\begin{array}{c} \mathrm{FRPS}=\beta 1\times \mathrm{Age}+\beta 2\times \mathrm{Sex}+\beta 3\times \mathrm{AF}+\beta 4\times \mathrm{HTN}+\beta 5\times \mathrm{DM}+\beta 6\times \mathrm{Hyperlipidemia}+\beta 7\times \mathrm{Cognitive}\;\mathrm{Impairment}\\ +\beta 8\times \mathrm{Pre}\text{-}\mathrm{Stroke}\;\mathrm{mRS}+\beta 9\times \mathrm{SBP}+\beta 10\times \mathrm{Onset}\text{-}\mathrm{to}\text{-}\mathrm{Puncture}\;\mathrm{Time}+\beta 11\times \mathrm{sICH}+\beta 12\times \mathrm{NIHSS}\;\mathrm{Score} \end{array}$$

where

*β*1, *β*2, …, *β*12 are the coefficients determined by the random forest model.

Age [10,11] is the age of the patient [10,11].

Sex is a binary variable representing the patient’s gender (e.g., male = 0, female = 1) [12].

Atrial fibrillation (AF) [11], hypertension (HTN), diabetes mellitus (DM) [11], hyperlipidemia [13,14], cognitive impairment [15,16], and symptomatic intracerebral hemorrhage (sICH) [17] are binary variables indicating each condition’s presence (1) or absence (0).

The pre-stroke mRS score represents the pre-stroke modified Rankin scale score [11].

SBP is the systolic blood pressure of the patient [17].

The onset-to-puncture time [10] is the interval from stroke onset to puncture during endovascular thrombectomy [10,11].

The NIHSS score is the National Institutes of Health Stroke Scale score [10,11].

As the random forest model automatically determines the coefficients during training, we do not have explicit coefficients as in linear regression. Instead, we used the random forest to calculate feature importance, which represents the contribution of each predictor variable to the prediction of the target variable (the FRPS score in this case). We extracted feature importance from the trained random forest model to determine the relative importance of each predictor variable (Supplemental Information S2 Online for Python code). The significance of these features provides insights into which variables have the most significant impact on FRPS score prediction.

## 3. Results

Based on the feature importance obtained from the modeling, the final equation for the FRPS model is

$$\begin{array}{c} \mathrm{FRPS}=(0.769\times \mathrm{Onset}\text{-}\mathrm{to}\text{-}\mathrm{Puncture}\;\mathrm{Time})+(0.140\times \mathrm{SBP})+(0.086\times \mathrm{Age})+(0.002\times \mathrm{NIHSS}\;\mathrm{Score})+(0.001\\ \times \mathrm{Pre}\text{-}\mathrm{Stroke}\;\mathrm{mRS})+(0.001\times \mathrm{DM})+(0.0005\times \mathrm{Hyperlipidemia})+(0.0005\times \mathrm{AF})+(0.0004\times \mathrm{Cognitive}\\ \mathrm{Impairment})+(0.0004\times \mathrm{HTN})+(0.0003\times \mathrm{Sex})+(0.0003\times \mathrm{sICH}) \end{array}$$

The random forest model achieved a mean R-squared value of approximately 0.992, indicating a strong relationship between the predictor variables and FRPS scores. We simulated FRPS scores for 1000 hypothetical patients and defined severity ranges based on percentiles. The ranges were classified as follows: mild (FRPS score < 66), moderate (FRPS score ≥ 66 and <81), and severe (FRPS score ≥ 81).

**Figure 1:**
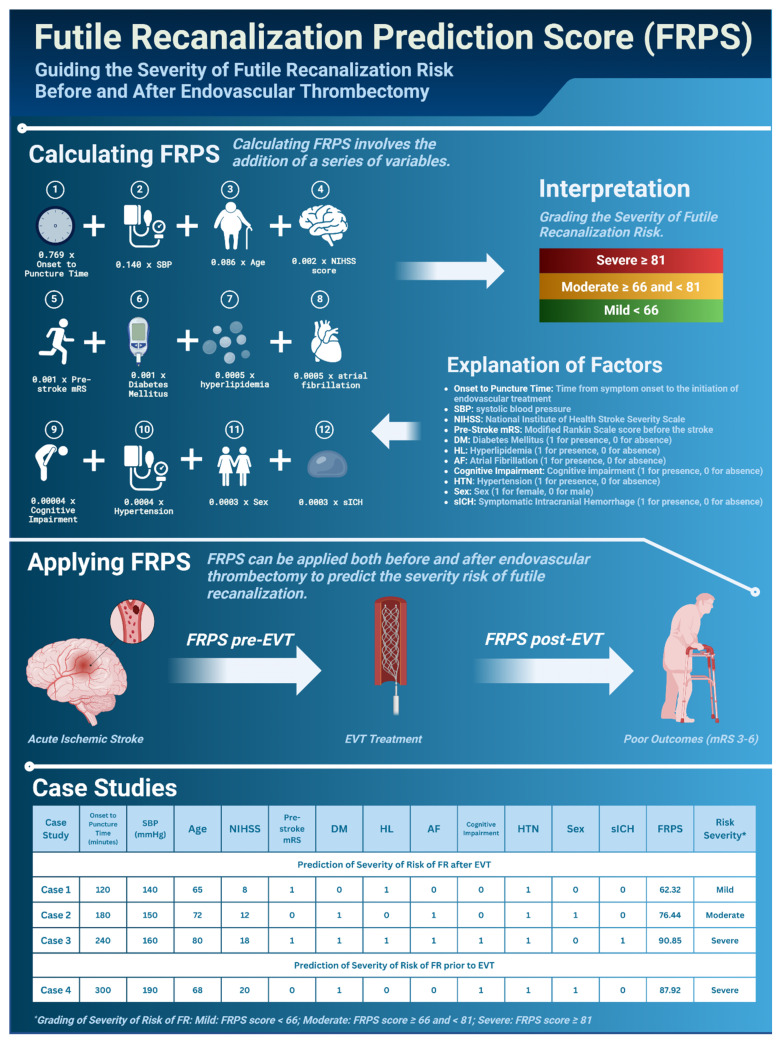
Utilization Schematic of the Futile Recanalization Prediction Score (FRPS) in a Clinical Setting. Abbreviations: FRPS: Futile Recanalization Prediction Score; FR: Futile Recanalization; SBP: Systolic Blood Pressure; NIHSS: National Institutes of Health Stroke Scale; mRS: Modified Rankin Scale; DM: Diabetes Mellitus; HL: Hyperlipidemia; AF: Atrial Fibrillation; HTN: Hypertension; sICH: Symptomatic Intracranial Hemorrhage; EVT: Endovascular Thrombectomy.

The FRPS can be applied in two settings (see Figure 1 for various case studies for illustrative purposes): to understand the risk of FR before EVT administration and to predict FR risk post-EVT. Future validation and refinement are necessary to ensure generalizability to diverse patient populations. Notably, for pre-EVT risk prediction, the anticipated time from symptom onset to puncture was presumed to be the current time since stroke onset at the risk calculation instance.

Figure 1 shows an example of the integration of this score within a clinical setting.

## 4. Discussion

**Figure 2:**
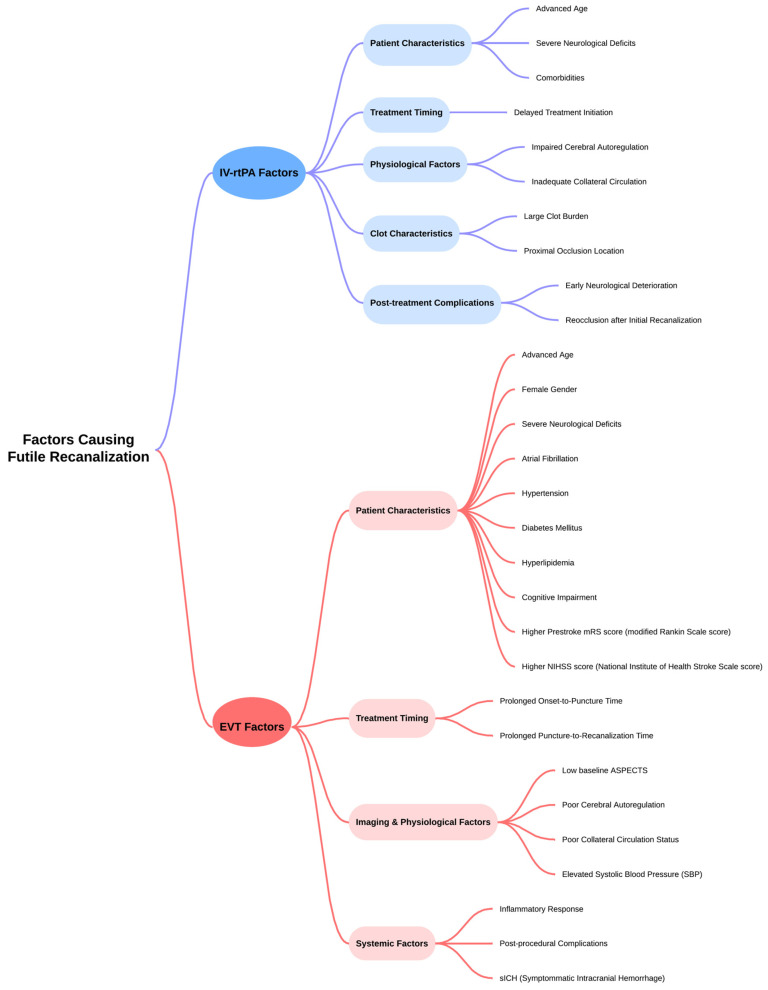
Factors Contributing to Futile Recanalization—A Flowchart Summary for IV-rtPA and EVT Treatments. Abbreviations: IV: Intravenous; rtPA: Recombinant Tissue Plasminogen Activator; EVT: Endovascular Thrombectomy; ASPECTS: Alberta Stroke Program Early Computed Tomography Score.

In this study, we propose a novel risk prediction score called the FRPS and demonstrate its high prognostic accuracy. We also discuss case studies for illustrative purposes (Figure 2).

### 4.1. Implications of Futile Recanalization and Clinical Need for Risk Prediction

The implications of FR and the need for risk prediction in clinical practice are significant for advancing personalized stroke care, particularly in the context of EVT. Understanding and predicting FR can yield multiple benefits:

***Recanalization Selection for EVT***: Identifying patients at high risk of FR can aid clinicians in making more informed decisions about the suitability of EVT versus alternative treatments.

***Tailoring Postprocedural Care****:* Patients who undergo EVT may require tailored postprocedural management strategies based on their FR risk. For example, high-risk patients may benefit from more intensive monitoring, early rehabilitation interventions, or aggressive secondary prevention measures, ultimately optimizing patient outcomes.

***Long-Term Treatment Planning***: Predicting FR likelihood informs the development of long-term treatment plans and helps manage expectations for therapeutic effects. Patients unlikely to achieve meaningful recanalization may necessitate ongoing surveillance, medication regimen adjustments, or exploration of alternative treatment options to prevent recurrent strokes and manage long-term disability.

Overall, the ability to understand and predict FR not only optimizes patient selection for EVT but also guides postprocedural care and long-term treatment strategies. This personalized approach has the potential to enhance clinical outcomes, optimize resource allocation, and improve the quality of life for patients with acute ischemic stroke and other vascular conditions.

### 4.2. Rationale and Development of a Futile Recanalization Risk Score

While EVT has shown effectiveness in improving outcomes for AIS patients, a significant portion does not benefit due to inadequate patient selection (Table 1) [10]. Despite successful recanalization, approximately 48.7% to 51% of patients fail to achieve favorable clinical outcomes. This shortfall may stem from relying solely on imaging criteria, such as identifying large vessel occlusion, without considering other prognostic factors. Therefore, a more comprehensive approach to patient selection is imperative, integrating imaging parameters and clinical variables such as age, comorbidities, and stroke severity (Figure 2).

Additionally, treatment processes, timing, and post-stroke physiological and systemic factors play vital roles [18,19,20]. This suggests the importance of incorporating advanced imaging modalities, such as perfusion imaging, to identify individuals likely to benefit from EVT. The FRPS provides a robust framework for predicting the risk of FR, potentially enhancing personalized treatment strategies and improving outcomes for AIS patients undergoing EVT.

Enhancing patient selection for EVT is critical to mitigating FR occurrence and improving outcomes for AIS patients [11]. FR is a pivotal aspect of AIS care, and prioritizing EVT for patients with the most significant potential benefit is essential, especially given the limited availability of this treatment. The efficacy of EVT varies among different AIS patient subgroups, with specific clinical and imaging characteristics potentially predisposing individuals to higher postprocedure FR rates [4,11,12,21]. Understanding the incidence, predictors, and consequences of FR in acute stroke patients is vital for assessing the appropriateness of interventions and facilitating tailored medical approaches, which are essential for optimizing results and minimizing complications in acute stroke management. This personalized approach not only aids in effectively managing AIS patients but also facilitates informed discussions regarding the risks and benefits of EVT procedures with patients and their families.

With advanced EVT devices, such as stent retrievers and aspiration catheters equipped with radio force adaptation, enhanced flexibility, and larger diameters, outcomes have vastly improved [22]. However, translating these advancements into routine clinical settings remains challenging due to substantial EVT procedure risks. The complexity of clot locations within intricate cerebral vasculature regions, such as the M2/3 arteries [23], increases the risk of complications and mortality rates, especially for patients with vulnerable atherosclerotic plaques [24]. This complexity heightens the risk of perforation, frequently resulting in postprocedural complications and increased mortality rates [24]. Moreover, patients with anatomical anomalies, such as an incomplete circle of Willis (CoW), face elevated mortality risks following EVT compared to those with a complete CoW [25]. Tandem occlusions, characterized by large vessel occlusions accompanied by significant stenosis exceeding 90%, further exacerbate the challenges encountered during EVT procedures for individuals affected by such pathologies [26].

In recent years, there has been a growing debate surrounding the baseline infarct volume thresholds predisposing individuals to FR following AIS and whether IVT offers protective benefits [27]. The volume of infarcted tissue holds significant importance in AIS management, as it directly correlates with the size of the penumbra, representing salvageable brain tissue at risk of infarction. Various studies have yielded conflicting results: some have indicated that IVT is a predictor of FR [8,28,29], while others have suggested that IVT may have protective effects [9,30,31,32]. Furthermore, some studies have reported the minimal impact of thrombolytic therapy on the development of FR [33,34,35,36,37]. Conflicting study results highlight the need for further comprehensive investigations to provide crucial evidence in this pivotal area of research.

Several recent RCTs examining EVT effectiveness for large core infarcts have shown improved functional outcomes and lower mortality rates compared to medical therapy alone [38,39,40,41,42,43]. However, patients experienced more intracranial hemorrhages [41], particularly those with lower ASPECTS scores of 3 or less [41]. Outcomes following FR require further elucidation, as FR is significantly associated with increased odds of adverse outcomes such as sICH, HT, and 90-day mortality. Anatomical anomalies, such as complex aortic arch and carotid artery anatomy, could also be potentially associated with procedural complications, thereby increasing the risk of adverse outcomes following FR [44]. Device or procedural complications may also contribute to this risk [45,46,47]. The impact of sICH on outcomes after FR is also an important consideration. Available evidence suggests that patients who experience FR after EVT during a stroke may be at an increased risk for sICH [48]. However, the relationship between FR and sICH may be complex, and other factors may also play a role in predicting sICH.

Efforts to minimize sICH risk should be prioritized in EVT management for stroke patients. Understanding the complexities surrounding EVT outcomes and associated risks is crucial for optimizing stroke care and patient outcomes. Further research, refining patient selection criteria, and mitigating postprocedure complications are imperative for advancing stroke treatment and enhancing patient care. Other mitigation strategies could include refining patient selection using advanced imaging, employing more efficient thrombectomy devices, and providing intensive postprocedural care. Exploring interventions and rehabilitation strategies is crucial for effectively addressing FR. Given its complexity, continued research into FR mechanisms and the development of innovative prevention and management strategies are warranted. Prioritizing these efforts is essential for advancing stroke care and improving functional outcomes in AIS patients undergoing EVT.

## 5. Conclusions

In conclusion, we have developed a novel Futile Recanalization Prediction Score (FRPS) to predict the severity risk of FR in patients eligible for or treated with EVT. Our detailed proposal includes mathematical modeling and simulations, which can help estimate FR risk pre- and post-EVT and assess procedural efficacy. However, further studies are needed to validate our scoring system and its applicability in diverse clinical settings. FR following EVT for AIS patients poses a significant challenge, potentially impacting patient outcomes and recovery trajectories even after a technically successful procedure. FR is associated with adverse effects such as sICH and reduced functional independence, substantially affecting the quality of life of stroke survivors. Strategies to address FR include refined patient selection through advanced imaging techniques, the utilization of more efficient thrombectomy devices to optimize clot removal, and rigorous postprocedural care to minimize complications. The introduction of the FRPS offers significant potential for advancing personalized medicine approaches to stroke care by improving prognostic accuracy and optimizing treatment planning. This tool represents a step forward in mitigating the risks associated with FR and enhancing overall outcomes for AIS patients.

## Figures and Tables

**Table 1:** Summary of the meta-analyses on futile recanalization after endovascular thrombectomy in acute ischemic stroke patients.

Author, y		Shahid et al., 2022 [8]	Deng et al., 2022 [9]	Shen et al., 2023 [7]
No. of studies	N	22	12	39
No. of patients	*n*	3037	2138	11,700
Search strategy until	-	February 2021	April 2021	May 2023
**FR prevalence**				
Prevalence	Percentage [95% CI; *p*-value]	51% [45.8–54.7%]	48.7% (only crude prevalence reported)	51% [48–54%; *p* < 0.001]
**FR predictors**				
Age	SMD [95% CI; *p*-value]	5.6 [4.7–6.6; *p* < 0.001]	5.81 [4.16–7.46; *p* < 0.00001]	0.49 [0.42–0.56; *p* < 0.0001]
AF	OR [95% CI; *p*-value]	1.5 [1.2–1.8; *p* < 0.001]	1.24 [1.01–1.51; *p* < 0.00001]	1.39 [1.22–1.59; *p* < 0.001]
Alcohol	OR [95% CI; *p*-value]	NR	NR	0.80 [0.581–1.101; *p* = 0.170]
CVD	OR [95% CI; *p*-value]	1.4 [1.1–1.8; *p* < 0.01]	NR	1.15 [0.795–1.671; *p* = 0.454]
HTN	OR [95% CI; *p*-value]	1.5 [1.3–1.9; *p* < 0.001]	1.73 [1.43–2.09; *p* < 0.00001]	1.65 [1.41–1.92; *p* < 0.001]
HL	OR [95% CI; *p*-value]	1.1 [0.9–1.3; *p* = 0.20]	1.01 [0.80–1.28; *p* = 0.92]	0.97 [0.870–1.088; *p* = 0.627]
DM	OR [95% CI; *p*-value]	1.5 [1.1–2.1; *p* = 0.1]	1.78 [1.41–2.24; *p* < 0.00001]	1.71 [1.47–1.99; *p* < 0.001]
Male sex	OR [95% CI; *p*-value]	NR	NR	0.87 [0.77–0.97; *p* = 0.016]
Female sex	OR [95% CI; *p*-value]	1.3 [1.1–1.6; *p* < 0.01]	1.40 [1.16–1.68; *p* < 0.0004]	NR
PS/TIA	OR [95% CI; *p*-value]	1.4 [1.03–2.04; *p* < 0.03]	NR	1.30 [1.06–1.59; *p* = 0.012]
Smoking	OR [95% CI; *p*-value]	0.6 [0.5–0.7; *p* < 0.01]	NR	0.66 [0.57–0.77; *p* < 0.001]
GC	OR [95% CI; *p*-value]	NR	NR	0.33 [0.23–0.49; *p* < 0.001]
APU	OR [95% CI; *p*-value]	1.1 [0.8–1.4; *p* = 0.58]	NR	1.16 [0.976–1.386; *p* = 0.094]
ACU	OR [95% CI; *p*-value]	0.5 [0.1–1.6; *p* = 0.23]	NR	1.33 [1.08–1.63; *p* = 0.007]
LAA	OR [95% CI; *p*-value]	NR	0.92 [0.70–1.21; *p* = 0.54]	0.83 [0.671–1.018; *p* = 0.073]
CE	OR [95% CI; *p*-value]	NR	1.06 [0.85–1.33; *p* = 0.60]	1.34 [1.10–1.63; *p* = 0.003]
GA	OR [95% CI; *p*-value]	1.2 [0.78–2.01; *p* = 0.34]	NR	1.53 [1.35–1.74; *p* < 0.001]
IVT	OR [95% CI; *p*-value]	0.7 [0.5–0.8; *p* < 0.001]	0.67 [0.55–0.83; *p* < 0.0001]	0.75 [0.66–0.86; *p* < 0.001]
BG	SMD [95% CI; *p*-value]	NR	0.59 [0.37–0.81; *p* < 0.00001]	0.31 [0.22–0.41; *p* < 0.001]
SBP	SMD [95% CI; *p*-value]	6.9 [3.6–8.7; *p* < 0.001]	4.98 [1.87–8.09; *p* < 0.002]	0.20 [0.13–0.27; *p* < 0.001]
DBP	SMD [95% CI; *p*-value]	1.31 [−1.0–3.6; *p* = 0.26]	−0.36 [−3.14–2.42; *p* = 0.80]	NR
NIHSS	SMD [95% CI; *p*-value]	4.2 [3.2–5.1; *p* < 0.001]	4.22 [3.38–5.07; *p* < 0.00001]	0.75 [0.65–0.86; *p* < 0.001]
ASPECTS	SMD [95% CI; *p*-value]	−0.5 [−0.8– −0.3; *p* < 0.001]	−0.71 [−1.23–−0.19; *p* = 0.007]	−0.37 [−0.46–−0.27; *p* < 0.001]
OTT	SMD [95% CI; *p*-value]	24.3 [9.9–38.7; *p* < 0.001]	16.92 [6.52–27.31; *p* < 0.001]	0.22 [0.13–0.30; *p* < 0.001]
PTR	SMD [95% CI; *p*-value]	9.58 [5.3–13.8; *p* < 0.001]	12.37 [7.96–16.79; *p* < 0.00001]	NR
OTR	SMD [95% CI; *p*-value]	32.1 [6.5–47.7; *p* < 0.001]	13.97 [−7.85–35.80; *p* = 0.21]	0.38 [0.19–0.57; *p* < 0.001]
OTED	SMD [95% CI; *p*-value]	20.1 [4.4–35.8; *p* < 0.01]	NR	NR
ICA occlusion	OR [95% CI; *p*-value]	NR	1.85 [1.17–2.95; *p* = 0.009]	NR
MCA-MI occlusion	OR [95% CI; *p*-value]	NR	0.81 [0.51–1.28; *p* = 0.37]	NR
MCA-M2 occlusion	OR [95% CI; *p*-value]	NR	0.70 [0.42–1.18; *p* = 0.19]	NR
Tandem occlusion	OR [95% CI; *p*-value]	NR	1.30 [0.72–2.33; *p* = 0.38]	NR
Procedure complications	OR [95% CI; *p*-value]	0.8 [0.4–1.8; *p* = 0.61]	NR	NR
**FR outcomes**				
sICH	OR [95% CI; *p*-value]	5.7 [2.8–11.65; *p* < 0.01]	6.09 [3.18–11.68; *p* < 0.00001]	7.37 [4.89–11.12; *p* < 0.001]
HT	OR [95% CI; *p*-value]	NR	NR	2.98 [2.37–3.75; *p* < 0.001]
90-day mortality	OR [95% CI; *p*-value]	NR	NR	19.24 [1.57–235.18; *p* = 0.021]
**Data processing and evaluation**				
Meta-regression	-	Applied	Applied	Applied
Sensitivity analysis	-	NP	NP	Applied
Trails sequential analysis	-	NP	NP	NP
Evidence of effect	-	NP	NP	NP

Abbreviations: FR = futile recanalization; SMD = standard mean difference; CI = confidence interval; AF = atrial fibrillation; OR = odds ratio; NR = not reported; CVD = cardiovascular disease; HTN = hypertension; HL = hyperlipidemia, DM = diabetes mellitus; PS/TIA = prior stroke/transient ischemic attack; GC = good collaterals; APU = antiplatelet usage; ACU = anticoagulant usage; LAA = large artery atherosclerosis; CE = cardioembolic; GA = general anesthesia; IVT = intravenous thrombolysis; BG = blood glucose; SBP = systolic blood pressure; DBP = diastolic blood pressure; NIHSS = National Institutes of Health Stroke Severity; ASPECTS = Alberta Stroke Program Early CT Score, OTT = onset-to-treatment time; PTR = puncture-to-recanalization time; OTR = onset-to-recanalization time; OTED = onset-to-emergency-department-arrival time; ICA = internal carotid artery; MCA = middle cerebral artery; sICH = symptomatic intracranial hemorrhage; HT = hemorrhagic transformation; NP = not performed.

## Data Availability

The original contributions presented in this study, including the Python codes to generate the model for the futile recanalization prediction score, are included in the study, as well as the online Supplementary Information. Further inquiries can be directed to the corresponding author.

## Supplementary Materials

The following supporting information can be downloaded at: https://www.mdpi.com/article/10.3390/neurolint16030045/s1, Supplemental Information S1 Background on Futile Recanalization: Definition, Prevalence, Predictors, and Outcomes after Futile Recanalization; Supplementary Information S2: Python Code. References [49,50,51,52,53,54,55,56,57,58,59,60,61,62,63,64,65,66,67,68,69,70,71,72,73,74,75,76,77,78,79,80,81,82,83,84,85,86,87,88,89,90,91,92,93,94,95,96,97,98,99,100,101,102,103,104,105,106,107,108,109,110,111,112,113,114,115,116,117,118,119,120] are cited in the Supplementary Materials.

## Abbreviations

**Abbreviation**	**Definition**
AF	Atrial Fibrillation
AHA	American Heart Association
ASA	American Stroke Association
ASPECTS	Alberta Stroke Program Early CT Score
BAO	Basilar Artery Occlusion
BBB	Blood–Brain Barrier
CMR	Clinically Meaningful Recanalization
CT	Computed Tomography
DALYs	Disability-Adjusted Life Years
DM	Diabetes Mellitus
EVT	Endovascular Thrombectomy
FR	Futile Recanalization
FRPS	Futile Recanalization Prediction Score
GA	General Anesthesia
GBD	Global Burden of Disease
HDMCA	Hyperdense Middle Cerebral Artery Sign
HL	Hyperlipidemia
HT	Hemorrhagic Transformation
ICU	Intensive Care Unit
IV-rtPA	Intravenous Recombinant Tissue Plasminogen Activator
IVT	Intravenous Thrombolysis
LVO	Large Vessel Occlusion
MCA	Middle Cerebral Artery
mRS	Modified Rankin Score
mTICI	Thrombolysis in Cerebral Infarction
NLR	Neutrophil-to-Lymphocyte Ratio
NIHSS	National Institutes of Health Stroke Severity Score
NNT	Number Needed to Treat
OTT	Onset-to-Treatment Time
OTR	Onset-to-Reperfusion Time
pc-ASPECTS	Posterior Circulation Alberta Stroke Program Early CT Score
PS/TIA	Previous Stroke/Transient Ischemic Attack
RCT	Randomized Control Trial
SMM	Standard Medical Management
sICH	Symptomatic Intracranial Hemorrhage
VHF	Vascular Hyperintensities on FLAIR Imaging
