# Supplementary material for: Introducing the Futile Recanalization Prediction Score (FRPS): A Novel Approach to Predict and Mitigate Ineffective Recanalization after Endovascular Treatment of Acute Ischemic Stroke
Source: Neurol Int. 2024 May 30;16(3):605–19. doi: 10.3390/neurolint16030045 (PMC11206671; doi:10.3390/neurolint16030045)
Supplement: Supplementary file 1 [file neurolint-16-00045-s001.zip › Supplemental_Information_1.pdf]

Supplemental Information 1

Introducing the Futile Recanalization Prediction Score (FRPS): A Novel Approach to Predict and Mitigate Ineffective Recanalization after Endovascular Treatment of Acute Ischemic Stroke

Supplemental Information 1: Background on Futile Recanalization: Definition, Prevalence, Predictors, and Outcomes after Futile Recanalization.

Endovascular Thrombectomy in Acute Ischemic Stroke

Endovascular thrombectomy (EVT) has emerged as a significant advancement in the treatment of acute ischemic stroke (AIS). In 2015, five randomized controlled trials (RCTs) demonstrated the superiority of EVT over standard medical management (SMM) in patients with anterior circulation stroke and proximal large vessel occlusion (LVO) [1-5]. A 2016 meta-analysis by the HERMES collaboration highlighted the time-sensitive nature of EVT, indicating that treatment within the first 7 hours poststroke is likely to yield the best outcomes [6]. Two meta-analyses were published in 2016, analyzing the functional outcomes of the 1287 patients from these RCTs through their scores on the modified Rankin score (mRS) scale at 90 days. Goyal *et al.* (2016) quantified the effectiveness of EVT and reported that the number needed to treat (NNT) with EVT to significantly reduce the mRS score by one level for one patient was 2.66 [1]. Additionally, Saver *et al.* (2016) reported that patients who underwent EVT and medical therapy had significantly lower rates of functional disability after 90 days than patients who underwent medical therapy alone [6]. Further RCTs, such as the DIRECT-MT and DEVT trials from China, demonstrated the noninferiority of EVT compared to combined therapy with intravenous alteplase[7]. The RESCUE-Japan LIMIT trial indicated that in the context of anterior circulation emergent large vessel occlusion (LVO) stroke, patients who presented with large ischemic cores, as indicated by lower scores on the Alberta Stroke Program Early CT Score (ASPECTS), experienced better functional outcomes when treated with EVT than when treated with SMM alone[8]. However, it is important to note that there was no significant difference in the likelihood of achieving an excellent functional outcome between the two treatment groups[9]. Overall, EVT is regarded as an effective and safe option for treating patients with large-core AIS, as determined by low ASPECTS or volumetric assessment methods [9]. The Merci device was recognized as the pioneering successful clot-retrieval device, demonstrating recanalization success in approximately half of the treated patients [10]. These advances together guided the development of the 2018 AHA/ASA Guidelines for the Early Management of Patients with AIS, which were further updated in 2021, with EVTs recommended as first-line treatment following IVT administration for AIS patients [11]. Current guidelines emphasize the importance of immediate brain imaging upon suspicion of AIS and recommend noninvasive intracranial vascular imaging for patients meeting EVT criteria or when LVO is suspected. Technique selection for EVT should be individualized, considering clinical characteristics and the technical performance of the procedure [12]. EVT can be performed using various techniques, including stent retrievers, aspirators, the Solumbra technique, and accessory devices [13]. The evidence from RCTs and meta-analyses, in conjunction with the AHA/ASA guidelines, highlights EVT's considerable benefits in treating AIS. The evolution of EVT techniques and the implementation of time-sensitive protocols have played pivotal roles in enhancing functional outcomes for AIS patients. Moreover, the emphasis on individualized technique selection and the prioritization of rapid imaging and treatment underscores the need for personalized and urgent approaches to AIS management.

Supplemental Information 1

Futile Recanalization

EVT is a widely utilized treatment for AIS caused by LVO [1]. Despite the high success rates of recanalization achieved through EVT, a significant proportion of patients may experience FR, characterized by the absence of significant clinical improvement despite successful restoration of blood flow [14]. FR indicates insignificant or minimal improvements in functional outcomes following EVT in AIS patients[13]. Successful recanalization is typically assessed through angiographic reperfusion outcome, the number of passes utilized during EVT, and the patient's functional outcome at 90 days[15]. Angiographic reperfusion outcomes are evaluated using the modified Thrombolysis In Cerebral Infarction (mTICI) score [16], while clinical functional outcomes at 90 days are assessed using the mRS. Earlier studies defined a successful recanalization procedure as achieving a TICI grade of 2b-3, indicating complete recanalization of the vascular territory, albeit potentially slower than usual filling, or antegrade flow into the vascular bed without any filling delay [16]. However, more recent studies have refined this definition, introducing a new subcategory of 2c within the TICI grading system. Full recanalization is now often defined as a reperfusion outcome of 2c-3, with the 2c grade representing near-complete perfusion of the ischemic territory [16]. This updated TICI grading system reflects a more nuanced understanding of recanalization and its relationship to functional outcomes. This evolution in criteria highlights ongoing efforts to enhance prognostic tools and improve patient care in the management of AIS.

Prevalence of Futile Recanalization

EVT represents a game-changing treatment for AIS patients [1]. Despite its significant advancement over previous treatment modalities [1], the persistence of FR remains a pressing concern in the landscape of reperfusion therapy, largely due to gaps in our understanding of its epidemiology [17]. **Table 1** offers an insight into various meta-analyses conducted on FR. A recent meta-analysis conducted by our group revealed a pooled prevalence estimate of 51%, with rates ranging from 48% to 54% [18], similar to findings from prior meta-analyses reporting similar pooled prevalence rates of 51% [19] and crude prevalence rates of 48.7% [20]. The variability in reported FR prevalence across studies highlights the challenge in identifying patients at risk of FR before EVT, potentially resulting in unnecessary procedures and missed opportunities for life-saving interventions [18-20]. Moreover, inconsistencies in FR definitions among studies may influence reported prevalence rates and predictors. Furthermore, several studies exploring FR predictors adopt a retrospective and observational approach, which may limit their capacity to establish causal relationships.

Geographical Variations

Geographical differences in reported FR rates raise concerns from a global health perspective [15,21,22]. These differences may stem from procedural variances or disparities in healthcare systems, but further investigation is needed to identify their underlying causes accurately. Moreover, the high prevalence of FR indicates its relatively common occurrence, particularly concerning given the recent incorporation of EVT into standard AIS care [11]. The variability in FR rates, coupled with the lack of clarity regarding its etiology and the observed regional discrepancies, underscores the importance of exploring FR prevalence more deeply. Our meta-analysis revealed geographical variabilities, with a prevalence of 53% in Asia, 48% in Europe, 62% in North America, 35% in the Middle East, and 40% in multinational studies encompassing various countries [18].

Predictors of Futile Recanalization

Identifying predictors of FR is important for refining patient selection and improving patient outcomes following EVT. Several studies have investigated the predictors of FR after EVT in stroke patients[23-26]. **Table 1** documents the predictors of FR as reported in previous meta-analyses. Key factors include age, gender, baseline stroke severity, and comorbidities. Other factors include imaging and physiological biomarkers, baseline collateral status, clot location, infarct size, onset-to-puncture time, IVT administration,

and the number of passes utilized during EVT. Understanding these predictors is vital for effectively managing AIS patients. Individuals with specific risk factors can be managed appropriately or, in select cases, may be exempt from EVT to minimize the likelihood of FR and associated risks. Resources can also be reserved for patients for whom the surgical procedure can yield optimal benefit.

Baseline Demographics

The effect of unmodifiable risk factors such as age and sex on the risk of developing FR is a topic of debate. While several studies have reported poorer outcomes in older AIS patients[18,19,27,28], particularly those above 80 years, following EVT compared to younger counterparts[22,29,30], clinical benefits of EVT are still evident in older age groups, even those over 85 years. Withholding EVT solely based on an individual's age is unjustified [31]. Further research is necessary to delineate age's precise impact on prognosis and provide better guidance for AIS patients and their families. Although some studies determined that female sex was associated with an increased risk of FR [18,32] and that male sex was protective [18], a meta-analysis by Chalos *et al.* (2019) [33] of 1762 patients revealed that women were no less susceptible to different clinical outcomes after EVT than men were. Further studies are required to better elucidate the exact effects of age and sex on prognosis, offering clearer guidance to AIS patients and their families.

Comorbidities and Stroke Severity

Preexisting comorbidities are also closely linked with the risk of FR following EVT. Comorbidities such as atrial fibrillation (AF) [18], hypertension [18,19,34], diabetes mellitus (DM) [18,22,28,34-36], previous stroke or transient ischemic attack (PS/TIA) [18], renal impairment [37,38], infective endocarditis [39], or prior anticoagulant usage [18] have been identified as possible predictors. Another predictor of FR is the stroke severity at admission. Our recent meta-analysis [18] also revealed that patients with more severe strokes, as measured by the baseline National Institutes of Health Stroke Scale (NIHSS), were more likely to experience FR. This finding is supported by multiple other studies [19,22,24,26,27,34,35,40,41].

Blood-based Biomarkers

Increased levels of creatinine are associated with FR [32]. Several blood-based biomarkers, including matrix metalloproteinase-9 (MMP-9), tenascin-C, thioredoxin, von Willebrand factor-cleaving protease (ADAMTS13), gelsolin, soluble tumor necrosis factor-like weak inducer of apoptosis (sTWEAK) and Cyastic C, are reportedly associated with poor outcomes following EVT or FR [42-44]. These biomarkers may contribute to inflammation, tissue remodeling, and coagulation processes, thereby mediating recovery after stroke. Inflammatory markers like low neutrophil-to-lymphocyte ratios (NLRs) and platelet-to-lymphocyte ratios (PLRs), as well as serum amyloid A, hold potential as prognostic indicators for outcomes after EVT [45,46]. However, several of these studies lack validation or replication, and their ability to predict FR warrants further investigation.

Imaging Biomarkers

The prognostic value of topographic imaging biomarkers, such as the Alberta Stroke Program Early CT Score (ASPECTS), originally designed to identify candidates for EVT[47], has garnered increasing interest [48]. These biomarkers, including the electronic ASPECTS (e-ASPECTS) systems, have shown promise not only in determining EVT eligibility but also in predicting the occurrence of FR [49]. Specifically, scores lower than 7[41], lower posterior ASPECTS (pc-ASEPCTS) [18,50], or pc-ASPECTS equal to or less than 8 [51], have been linked to FR. Other imaging biomarkers, such as hyperdense middle cerebral artery sign (HDMCA) and poorly extended vascular hyperintensities on fluid-attenuated inversion recovery (FLAIR) (VHF) imaging, defined by a VHF grade <3 at admission, have shown associations with FR[34,52]. Moreover, while less well-

understood, imaging-based biomarkers such as brain atrophy [53,54], leukoaraiosis [52,55,56], and cerebral microbleeds [55] also hold the potential for predicting FR occurrence.

Clot Location and Collateral Status

Clot location and baseline collateral status are important considerations, as they may mediate outcomes after EVT, especially FR [15,18,20,57]. As distal clots in the M3 region of the middle cerebral artery (MCA) and terminal internal carotid arteries are difficult to access through a stent, they are associated with poorer outcomes after EVT [58,59]. This may also be attributed to relatively fewer or minimal collateral arteries around the terminal branches of the ICA. However, other studies found minimal effects of collateral status on the development of FR [60]. Interestingly, collateral status has been found to be independent of FR if EVT is initiated within 6.5 hours of the first stroke symptoms [61]. Pooled data from the Mechanical Embolus Removal in Cerebral Ischemia (MERCI) and Multi MERCI trials reported higher rates of revascularization in AIS patients with isolated M2 occlusions compared to those with M1 occlusions (82.1% vs 60%) [62]. In addition, fewer passes and shorter median procedural times were required for M2 occlusions than for M1 occlusions. However, these early reperfusion outcomes did not translate to significant differences in long-term functional outcomes or mortality [62]. Moreover, the heterogeneity in catheters used during EVT may impact treatment and reperfusion outcomes. A recent study involving stroke patients and mice demonstrated the critical role of leptomeningeal collaterals in regulating reperfusion dynamics post-stroke [63]. By actively managing blood flow recovery, these collaterals play a critical role in preventing FR [64].

Reperfusion Time

The time elapsed from symptom onset to reperfusion treatment is a critical factor from a prognostic standpoint. Several key factors merit consideration:

Onset-to-treatment (OTT) time as a predictor: OTT is a significant predictor of FR [65-67]. Higher OTT and onset-to-reperfusion (OTR) times are linked to an elevated risk of FR [25],[18]. The time gap between OTT and OTR for rapid reperfusion is also linked to an increased risk of FR.

Protective role of faster OTT: Reduced OTT is protective against FR, notably within 90 minutes of attaining excellent reperfusion (mTICI grade 3) [66]. Timely intervention is crucial for successful recanalization and improved patient outcomes. Several cohort studies support the hypothesis that a shorter OTT is protective against FR [19,35,51,68-70], especially if the time is less than 90 minutes, and achieves mTICI-3 grade reperfusion [66]. However, one study found no association between OTT, lesion location, and FR[32], possibly due to using EVT onset time instead of reperfusion time [32].

Procedural Factors

Procedural factors such as the number of passes and the thrombectomy technique utilized have also been found to be related to outcomes after recanalization. Several studies have shown that recanalization with fewer passes is associated with better postprocedural outcomes [22,58,71-74], which can be achieved more frequently with balloon-guided catheters [58]. Studies have shown that 5 or more passes using a stent retriever [70,75] or aspiration thrombectomy are associated with an increased incidence of FR [65], indicating that those with lower baseline stroke severity are more likely to benefit from treatment even if more than 5 passes are utilized and that using more than 5 passes still leads to substantial functional benefits compared with stopping the passes after reaching 4 [76]. Aspiration and balloon-catheter methods were also more efficacious than stent retriever methods, analogous to findings from previous studies[58]. These discrepancies could be due to differing stroke profiles, for example, in terms of location, composition, and individual anatomical factors such as collateral circulation [76]. The exact technique the operator uses could also be subject to individual variability, and the amount of reperfusion needed to achieve good outcomes (mTICI2 or mTICI-3) is also debated [66]. Furthermore, few studies have yet to explicitly report FR, describing only the link between EVT techniques and poorer outcomes. However, further studies are

needed to elucidate better the exact effect of the number of passes on futile recanalization. Our recent meta-analysis also revealed that the use of general anesthesia (GA) during EVT is a predictor of FR [18].

Outcomes after Futile Recanalization

Patients who experience FR have worse outcomes than those who experience successful recanalization. FR can result in various adverse outcomes, most commonly worsening functional outcomes, defined using the mRS scale, mortality, and symptomatic intracranial hemorrhage (sICH). FR is significantly associated with increased odds of sICH, hemorrhagic transformation (HT), and 90-day mortality [18]. These outcomes can be a major impediment to prognosis and can lead to lifelong disability in patients. It is, hence, crucial for physicians to anticipate their occurrence so that patients and families can be accurately informed of the risks of undergoing EVTs.

Functional Deficits

Several studies [14,25,30,59,67,69,77,78] have reported the persistence of significant neurological deficits despite successful recanalization after EVT in stroke patients. One of the most important outcomes after FR is the patient's functional status, with studies showing that patients who experienced FR had significantly worse functional outcomes than those who experienced successful recanalization [25]. Other identified outcomes following FR were early neurological deterioration [14,42], neurological progression [79], brain herniation [42], parenchymal hypodensities or local brain swelling [42], 24-hour NIHSS changes [80,81], trail-making at 90 days [14], and distal clot migration [82], but further and more detailed studies are needed to gauge their relationship with FR better.

Hemorrhagic Transformations

Hemorrhagic transformation (HT) is a common complication following EVT for AIS. This condition occurs when the blood-brain barrier (BBB) is disrupted, leading to various types of hemorrhages within the brain[83]. sICH is one such type of HT, and its incidence may be influenced by the occurrence of FR[18]. Several studies have investigated the incidence of sICH after FR, revealing that the incidence of sICH was greater in patients who experienced FR than in those who experienced successful recanalization, revealing that the incidence of sICH was greater in patients who experienced FR [1,15,30,67,84] and suggesting that patients who experience FR may be at increased risk for sICH. However, the relationship between FR and sICH may be more complex than a simple cause-and-effect relationship. Factors other than recanalization status, such as age and initial stroke severity, may be more important predictors of sICH. Other outcomes include hemorrhagic infarction [85], subarachnoid hemorrhage [85,86], parenchymal hematomas [85,87], trail-making at 90 days [14], distal clot migration [82], and asymptomatic intracranial hemorrhage[14,88]. Further research is needed to understand the full spectrum of EVT risks and identify which patients may be more susceptible to complications such as sICH.

Mortality

One crucial outcome following FR is mortality. Several studies have demonstrated that patients experiencing FR face higher mortality rates compared to those achieving successful recanalization [23] [77] [15,18-20]. This raises questions about the comparative effectiveness of different techniques and emphasizes the importance of further research in this domain.

Environmental Outcomes

Factors such as the total duration of hospitalization, length of stay in the intensive care unit (ICU), need for intubation (especially beyond 7 hours post-stroke onset), and discharge destination are vital clinical considerations associated with FR [14,89]. However, reporting practices for these outcomes vary considerably across studies. The discharge destination of a patient is influenced by stroke severity and the effectiveness of reperfusion therapy [90]. These environmental outcomes are linked to increased healthcare

costs and resource utilization [14]. In addition, intubation and prolonged ICU stays can contribute to extended hospitalizations and poorer long-term prognosis. Despite limited data on sICH and 90-day mortality rates across multiple studies, available evidence suggests that FR is associated with worse functional outcomes and higher mortality rates compared to successful recanalization [56,69,78,91-94].

Supplemental References

1. Goyal, M.; Menon, B.K.; van Zwam, W.H.; Dippel, D.W.J.; Mitchell, P.J.; Demchuk, A.M.; Dávalos, A.; Majoie, C.B.L.M.; van der Lugt, A.; de Miquel, M.A.; et al. Endovascular thrombectomy after large-vessel ischaemic stroke: a meta-analysis of individual patient data from five randomised trials. *The Lancet* **2016**, *387*, 1723-1731, doi:10.1016/S0140-6736(16)00163-X.
2. Berkhemer, O.A.; Fransen, P.S.; Beumer, D.; van den Berg, L.A.; Lingsma, H.F.; Yoo, A.J.; Schonewille, W.J.; Vos, J.A.; Nederkoorn, P.J.; Wermer, M.J.; et al. A randomized trial of intraarterial treatment for acute ischemic stroke. *New England Journal of Medicine* **2015**, *372*, 11-20, doi:10.1056/NEJMoa1411587.
3. Campbell, B.C.; Mitchell, P.J.; Kleinig, T.J.; Dewey, H.M.; Churilov, L.; Yassi, N.; Yan, B.; Dowling, R.J.; Parsons, M.W.; Oxley, T.J.; et al. Endovascular therapy for ischemic stroke with perfusion-imaging selection. *New England Journal of Medicine* **2015**, *372*, 1009-1018, doi:10.1056/NEJMoa1414792.
4. Saver, J.L.; Goyal, M.; Bonafe, A.; Diener, H.C.; Levy, E.I.; Pereira, V.M.; Albers, G.W.; Cognard, C.; Cohen, D.J.; Hacke, W.; et al. Stent-retriever thrombectomy after intravenous t-PA vs. t-PA alone in stroke. *New England Journal of Medicine* **2015**, *372*, 2285-2295, doi:10.1056/NEJMoa1415061.
5. Jovin, T.G.; Chamorro, A.; Cobo, E.; de Miquel, M.A.; Molina, C.A.; Rovira, A.; San Román, L.; Serena, J.; Abilleira, S.; Ribó, M.; et al. Thrombectomy within 8 hours after symptom onset in ischemic stroke. *New England Journal of Medicine* **2015**, *372*, 2296-2306, doi:10.1056/NEJMoa1503780.
6. Saver, J.L.; Goyal, M.; van der Lugt, A.; Menon, B.K.; Majoie, C.B.; Dippel, D.W.; Campbell, B.C.; Nogueira, R.G.; Demchuk, A.M.; Tomasello, A.; et al. Time to Treatment With Endovascular Thrombectomy and Outcomes From Ischemic Stroke: A Meta-analysis. *JAMA* **2016**, *316*, 1279-1288, doi:10.1001/jama.2016.13647.
7. Wassélius, J.; Arnberg, F.; von Euler, M.; Wester, P.; Ullberg, T. Endovascular thrombectomy for acute ischemic stroke. *Journal of Internal Medicine* **2022**, *291*, 303-316, doi:10.1111/joim.13425.
8. Yoshimura, S.; Sakai, N.; Yamagami, H.; Uchida, K.; Beppu, M.; Toyoda, K.; Matsumaru, Y.; Matsumoto, Y.; Kimura, K.; Takeuchi, M.; et al. Endovascular Therapy for Acute Stroke with a Large Ischemic Region. *New England Journal of Medicine* **2022**, *386*, 1303-1313, doi:10.1056/NEJMoa2118191.
9. Lina, P.; Amrou, S.; Apostolos, S.; Georgios, M.; Robin, L.; Else Charlotte, S.; Guillaume, T.; Marios, P.; Georgios, T. Endovascular treatment for large-core ischaemic stroke: a meta-analysis of randomised controlled clinical trials. *Journal of Neurology, Neurosurgery & Psychiatry* **2023**, *94*, 781, doi:10.1136/jnnp-2023-331513.
10. Smith, W.S.; Sung, G.; Starkman, S.; Saver, J.L.; Kidwell, C.S.; Gobin, Y.P.; Lutsep, H.L.; Nesbit, G.M.; Grobelny, T.; Rymer, M.M.; et al. Safety and Efficacy of Mechanical Embolectomy in Acute Ischemic Stroke. *Stroke* **2005**, *36*, 1432-1438, doi:10.1161/01.STR.0000171066.25248.1d.
11. Kleindorfer, D.O.; Towfighi, A.; Chaturvedi, S.; Cockroft, K.M.; Gutierrez, J.; Lombardi-Hill, D.; Kamel, H.; Kernan, W.N.; Kittner, S.J.; Leira, E.C.; et al. 2021 Guideline for the Prevention of Stroke in Patients With Stroke and Transient Ischemic Attack: A Guideline From the American Heart Association/American Stroke Association. *Stroke* **2021**, *52*, e364-e467, doi:10.1161/str.0000000000000375.
12. Bathla, G.; Ajmera, P.; Mehta, P.M.; Benson, J.C.; Derdeyn, C.P.; Lanzino, G.; Agarwal, A.; Brinjikji, W. Advances in Acute Ischemic Stroke Treatment: Current Status and Future Directions. *AJNR Am J Neuroradiol* **2023**, *44*, 750-758, doi:10.3174/ajnr.A7872.
13. Chartrain, A.G.; Awad, A.J.; Mascitelli, J.R.; Shoirah, H.; Oxley, T.J.; Feng, R.; Gallitto, M.; De Leacy, R.; Fifi, J.T.; Kellner, C.P. Novel and emerging technologies for endovascular thrombectomy. *Neurosurg Focus* **2017**, *42*, E12, doi:10.3171/2017.1.Focus16518.
14. Hussein, H.M.; Saleem, M.A.; Qureshi, A.I. Rates and predictors of futile recanalization in patients undergoing endovascular treatment in a multicenter clinical trial. *Neuroradiology* **2018**, *60*, 557-563, doi:10.1007/s00234-018-2016-2.

15. Ni, H.; Wang, B.; Hang, Y.; Liu, S.; Jia, Z.-Y.; Shi, H.-B.; Zhao, L.-B. Predictors of Futile Recanalization in Patients with Intracranial Atherosclerosis-Related Stroke Undergoing Endovascular Treatment. *World Neurosurgery* **2023**, *171*, e752-e759, doi:10.1016/j.wneu.2022.12.101.
16. Fugate, J.E.; Klunder, A.M.; Kallmes, D.F. What is meant by "TICI"? *American Journal of Neuroradiology* **2013**, *34*, 1792-1797, doi:10.3174/ajnr.A3496.
17. Adcock, A.K.; Schwamm, L.H.; Smith, E.E.; Fonarow, G.C.; Reeves, M.J.; Xu, H.; Matsouaka, R.A.; Xian, Y.; Saver, J.L. Trends in Use, Outcomes, and Disparities in Endovascular Thrombectomy in US Patients With Stroke Aged 80 Years and Older Compared With Younger Patients. *JAMA Netw Open* **2022**, *5*, e2215869, doi:10.1001/jamanetworkopen.2022.15869.
18. Shen, H.; Killingsworth, M.C.; Bhaskar, S.M.M. Futile Recanalization After Endovascular Thrombectomy for Acute Ischemic Stroke: A Comprehensive Meta-Analysis of Prevalence, Predictive Markers, and Clinical Outcomes. *Life* **2023**, *13*, doi:10.3390/life13101965.
19. Shahid, A.H.; Abbasi, M.; Larco, J.L.A.; Madhani, S.I.; Liu, Y.; Brinjikji, W.; Savastano, L.E. Risk Factors of Futile Recanalization Following Endovascular Treatment in Patients With Large-Vessel Occlusion: Systematic Review and Meta-Analysis. *Stroke: Vascular and Interventional Neurology* **2022**, *2*, e000257, doi:10.1161/SVIN.121.000257.
20. Deng, G.; Xiao, J.; Yu, H.; Chen, M.; Shang, K.; Qin, C.; Tian, D.-S. Predictors of futile recanalization after endovascular treatment in acute ischemic stroke: a meta-analysis. *Journal of NeuroInterventional Surgery* **2022**, *14*, 881, doi:10.1136/neurintsurg-2021-017963.
21. Sohn, J.-H.; Kim, C.; Lee, M.; Kim, Y.; Jung Mo, H.; Yu, K.-H.; Lee, S.-H. Effects of prior antiplatelet use on futile reperfusion in patients with acute ischemic stroke receiving endovascular treatment. *European Stroke Journal* **2022**, 23969873221144814, doi:10.1177/23969873221144814.
22. Linfante, I.; Starosciak, A.K.; Walker, G.R.; Dabus, G.; Castonguay, A.C.; Gupta, R.; Sun, C.H.; Martin, C.; Holloway, W.E.; Mueller-Kronast, N.; et al. Predictors of poor outcome despite recanalization: a multiple regression analysis of the NASA registry. *Journal of NeuroInterventional Surgery* **2016**, *8*, 224-229, doi:10.1136/neurintsurg-2014-011525.
23. Brinjikji, W.; Lanzino, G. Regarding: "Localized Marked Elongation of the Distal Internal Carotid Artery with or without PHACE Syndrome: Segmental Dolichoectasia of the Distal Internal Carotid Artery". *American Journal of Neuroradiology* **2018**, *39*, E95, doi:10.3174/ajnr.A5686.
24. Broeg-Morvay, A.; Mordasini, P.; Bernasconi, C.; Bühlmann, M.; Pult, F.; Arnold, M.; Schroth, G.; Jung, S.; Mattle, H.P.; Gralla, J.; et al. Direct Mechanical Intervention Versus Combined Intravenous and Mechanical Intervention in Large Artery Anterior Circulation Stroke. *Stroke* **2016**, *47*, 1037-1044, doi:10.1161/STROKEAHA.115.011134.
25. Froehler, M.T.; Saver, J.L.; Zaidat, O.O.; Jahan, R.; Aziz-Sultan, M.A.; Klucznik, R.P.; Haussen, D.C.; Hellinger, F.R., Jr.; Yavagal, D.R.; Yao, T.L.; et al. Interhospital Transfer Before Thrombectomy Is Associated With Delayed Treatment and Worse Outcome in the STRATIS Registry (Systematic Evaluation of Patients Treated With Neurothrombectomy Devices for Acute Ischemic Stroke). *Circulation* **2017**, *136*, 2311-2321, doi:10.1161/circulationaha.117.028920.
26. Kim, J.-M.; Bae, J.-H.; Park, K.-Y.; Lee, W.J.; Byun, J.S.; Ahn, S.-W.; Shin, H.-W.; Han, S.-H.; Yoo, I.-H. Incidence and mechanism of early neurological deterioration after endovascular thrombectomy. *Journal of Neurology* **2019**, *266*, 609-615, doi:10.1007/s00415-018-09173-0.
27. Lattanzi, S.; Norata, D.; Divani, A.A.; Di Napoli, M.; Broggi, S.; Rocchi, C.; Ortega-Gutierrez, S.; Mansueto, G.; Silvestrini, M. Systemic Inflammatory Response Index and Futile Recanalization in Patients with Ischemic Stroke Undergoing Endovascular Treatment. *Brain Sciences* **2021**, *11*, doi:10.3390/brainsci11091164.
28. Ritvonen, J.; Sairanen, T.; Silvennoinen, H.; Virtanen, P.; Salonen, O.; Lindsberg, P.J.; Strbian, D. Comatose With Basilar Artery Occlusion: Still Odds of Favorable Outcome With Recanalization Therapy. *Frontiers in Neurology* **2021**, *12*, doi:10.3389/fneur.2021.665317.
29. Malhotra, A.; Wu, X.; Payabvash, S.; Matouk, C.C.; Forman, H.P.; Gandhi, D.; Sanelli, P.; Schindler, J. Comparative Effectiveness of Endovascular Thrombectomy in Elderly Stroke Patients. *Stroke* **2019**, *50*, 963-969, doi:10.1161/strokeaha.119.025031.
30. Hilditch, C.A.; Nicholson, P.; Murad, M.H.; Rabinstein, A.; Schaafsma, J.; Pikula, A.; Krings, T.; Pereira, V.M.; Agid, R.; Brinjikji, W. Endovascular Management of Acute Stroke in the Elderly: A Systematic Review and Meta-Analysis. *American Journal of Neuroradiology* **2018**, *39*, 887-891, doi:10.3174/ajnr.A5598.

31. McDonough, R.V.; Ospel, J.M.; Campbell, B.C.V.; Hill, M.D.; Saver, J.L.; Dippel, D.W.J.; Demchuk, A.M.; Majoie, C.; Brown, S.B.; Mitchell, P.J.; et al. Functional Outcomes of Patients ≥85 Years With Acute Ischemic Stroke Following EVT: A HERMES Substudy. *Stroke* **2022**, *53*, 2220-2226, doi:10.1161/strokeaha.121.037770.
32. Lee, S.-H.; Kim, B.J.; Han, M.-K.; Park, T.H.; Lee, K.B.; Lee, B.-C.; Yu, K.-H.; Oh, M.S.; Cha, J.K.; Kim, D.-H.; et al. Futile reperfusion and predicted therapeutic benefits after successful endovascular treatment according to initial stroke severity. *BMC Neurology* **2019**, *19*, 11, doi:10.1186/s12883-019-1237-2.
33. Chalos, V.; de Ridder, I.R.; Lingsma, H.F.; Brown, S.; van Oostenbrugge, R.J.; Goyal, M.; Campbell, B.C.V.; Muir, K.W.; Guillemin, F.; Bracard, S.; et al. Does Sex Modify the Effect of Endovascular Treatment for Ischemic Stroke? *Stroke* **2019**, *50*, 2413-2419, doi:10.1161/strokeaha.118.023743.
34. Bani-Sadr, A.; Escande, R.; Mechtouff, L.; Pavie, D.; Hermier, M.; Derex, L.; Choc, T.-H.; Eker, O.F.; Nighoghossian, N.; Berthezène, Y. Vascular hyperintensities on baseline FLAIR images are associated with functional outcome in stroke patients with successful recanalization after mechanical thrombectomy. *Diagnostic and Interventional Imaging* **2023**, doi:10.1016/j.diii.2023.02.005.
35. Dhillon, P.S.; Butt, W.; Marei, O.; Podlasek, A.; McConachie, N.; Lenthall, R.; Nair, S.; Malik, L.; Bhogal, P.; Makalanda, H.L.D.; et al. Incidence and predictors of poor functional outcome despite complete recanalisation following endovascular thrombectomy for acute ischaemic stroke. *Journal of Stroke and Cerebrovascular Diseases* **2023**, *32*, 107083, doi:10.1016/j.jstrokecerebrovasdis.2023.107083.
36. Bradley, S.A.; Smokovski, I.; Bhaskar, S.M.M. Impact of diabetes on clinical and safety outcomes in acute ischemic stroke patients receiving reperfusion therapy: A meta-analysis. *Adv Clin Exp Med* **2022**, *31*, 583-596, doi:10.17219/acem/146273.
37. Nam, H.S.; Kim, B.M. Advance of Thrombolysis and Thrombectomy in Acute Ischemic Stroke. *J Clin Med* **2023**, *12*, doi:10.3390/jcm12020720.
38. Wang, R.; Xie, Z.; Li, B.; Zhang, P. Renal impairment and the prognosis of endovascular thrombectomy: a meta-analysis and systematic review. *Ther Adv Neurol Disord* **2022**, *15*, 17562864221083620, doi:10.1177/17562864221083620.
39. Maheshwari, R.; Cordato, D.J.; Wardman, D.; Thomas, P.; Bhaskar, S.M.M. Clinical outcomes following reperfusion therapy in acute ischemic stroke patients with infective endocarditis: a systematic review. *J Cent Nerv Syst Dis* **2022**, *14*, 11795735221081597, doi:10.1177/11795735221081597.
40. Brott, T.; Adams, H.P., Jr.; Olinger, C.P.; Marler, J.R.; Barsan, W.G.; Biller, J.; Spilker, J.; Holleran, R.; Eberle, R.; Hertzberg, V.; et al. Measurements of acute cerebral infarction: a clinical examination scale. *Stroke* **1989**, *20*, 864-870, doi:10.1161/01.str.20.7.864.
41. Zhang, Y.; Zhang, L.; Zhang, Y.; Li, Z.; Zhang, Y.; Xing, P.; Chen, W.; Wang, S.; Li, T.; Yang, P.; et al. Endovascular Recanalization for Acute Internal Carotid Artery Terminus Occlusion: A Subgroup Analysis From the Direct-MT Trial. *Neurosurgery* **2022**, *91*, 596-603, doi:10.1227/neu.0000000000002085.
42. Zang, N.; Lin, Z.; Huang, K.; Pan, Y.; Wu, Y.; Wu, Y.; Wang, S.; Wang, D.; Ji, Z.; Pan, S. Biomarkers of Unfavorable Outcome in Acute Ischemic Stroke Patients with Successful Recanalization by Endovascular Thrombectomy. *Cerebrovascular Diseases* **2020**, *49*, 583-592, doi:10.1159/000510804.
43. Su, M.; Zhou, Y.; Chen, Z.; Pu, M.; Li, Z.; Du, H.; Xu, G. Cystatin C predicts futile recanalization in patients with acute ischemic stroke after endovascular treatment. *Journal of Neurology* **2022**, *269*, 966-972, doi:10.1007/s00415-021-10680-w.
44. Hervella, P.; Sampedro-Viana, A.; Rodríguez-Yáñez, M.; López-Dequidt, I.; Pumar, J.M.; Mosqueira, A.J.; Fernández-Rodicio, S.; Bazarra-Barreiros, M.; Serena, J.; Silva-Blas, Y.; et al. Systemic biomarker associated with poor outcome after futile reperfusion. *Eur J Clin Invest* **2024**, e14181, doi:10.1111/eci.14181.
45. Liao, J.s.; Guo, C.; Zhang, B.; Yang, J.; Zi, W.; Li, J.l. Low neutrophil-to-lymphocyte and platelet-to-lymphocyte ratios predict favorable outcomes after endovascular treatment in acute basilar artery occlusion: subgroup analysis of the BASILAR registry. *BMC Neurology* **2023**, *23*, 113, doi:10.1186/s12883-023-03161-2.
46. Yeh, S.-J.; Chen, C.-H.; Lin, Y.-H.; Tsai, L.-K.; Lee, C.-W.; Tang, S.-C.; Jeng, J.-S. Serum amyloid A predicts poor functional outcome in patients with ischemic stroke receiving endovascular thrombectomy: a case control study. *Journal of NeuroInterventional Surgery* **2023**, *15*, 75, doi:10.1136/neurintsurg-2021-018234.
47. Ryu, C.W.; Shin, H.S.; Park, S.; Suh, S.H.; Koh, J.S.; Choi, H.Y. Alberta Stroke Program Early CT Score in the Prognostication after Endovascular Treatment for Ischemic Stroke: A Meta-analysis. *Neurointervention* **2017**, *12*, 20-30, doi:10.5469/neuroint.2017.12.1.20.

48. Liu, D.; Scalzo, F.; Rao, N.M.; Hinman, J.D.; Kim, D.; Ali, L.K.; Saver, J.L.; Sun, W.; Dai, Q.; Liu, X.; et al. Fluid-Attenuated Inversion Recovery Vascular Hyperintensity Topography, Novel Imaging Marker for Revascularization in Middle Cerebral Artery Occlusion. *Stroke* **2016**, *47*, 2763-2769, doi:10.1161/strokeaha.116.013953.
49. Pfaff, J.; Herweh, C.; Schieber, S.; Schönenberger, S.; Bösel, J.; Ringleb, P.A.; Möhlenbruch, M.; Bendszus, M.; Nagel, S. e-ASPECTS Correlates with and Is Predictive of Outcome after Mechanical Thrombectomy. *American Journal of Neuroradiology* **2017**, *38*, 1594-1599, doi:10.3174/ajnr.A5236.
50. Lu, W.Z.; Lin, H.A.; Bai, C.H.; Lin, S.F. Posterior circulation acute stroke prognosis early CT scores in predicting functional outcomes: A meta-analysis. *PLoS ONE* **2021**, *16*, e0246906, doi:10.1371/journal.pone.0246906.
51. Ouyang, K.; Kang, Z.; Liu, Z.; Hou, B.; Fang, J.; Xie, Y.; Liu, Y. Posterior Circulation ASPECTS on CT Angiography Predicts Futile Recanalization of Endovascular Thrombectomy for Acute Basilar Artery Occlusion. *Frontiers in Neurology* **2022**, *13*, doi:10.3389/fneur.2022.831386.
52. Karatzetzou, S.; Tsiptsios, D.; Sousanidou, A.; Christidi, F.; Psatha, E.A.; Chatzaki, M.; Kitmeridou, S.; Giannakou, E.; Karavasilis, E.; Kokkotis, C.; et al. Elucidating the Role of Baseline Leukoaraiosis on Forecasting Clinical Outcome of Acute Ischemic Stroke Patients Undergoing Reperfusion Therapy. *Neurol Int* **2022**, *14*, 923-942, doi:10.3390/neurolint14040074.
53. Pedraza, M.I.; de Lera, M.; Bos, D.; Calleja, A.I.; Cortijo, E.; Gómez-Vicente, B.; Reyes, J.; Coco-Martín, M.B.; Calonge, T.; Agulla, J.; et al. Brain Atrophy and the Risk of Futile Endovascular Reperfusion in Acute Ischemic Stroke. *Stroke* **2020**, *51*, 1514-1521, doi:10.1161/strokeaha.119.028511.
54. Diprose, W.K.; Diprose, J.P.; Wang, M.T.M.; Tarr, G.P.; McFetridge, A.; Barber, P.A. Automated Measurement of Cerebral Atrophy and Outcome in Endovascular Thrombectomy. *Stroke* **2019**, *50*, 3636-3638, doi:10.1161/strokeaha.119.027120.
55. Xu, T.; Wang, Y.; Yuan, J.; Chen, Y.; Luo, H. Small Vessel Disease Burden and Outcomes of Mechanical Thrombectomy in Ischemic Stroke: A Systematic Review and Meta-Analysis. *Frontiers in Neurology* **2021**, *12*, 602037, doi:10.3389/fneur.2021.602037.
56. Gilberti, N.; Gamba, M.; Premi, E.; Costa, A.; Vergani, V.; Delrio, I.; Spezi, R.; Dikran, M.; Frigerio, M.; Gasparotti, R.; et al. Leukoaraiosis is a predictor of futile recanalization in acute ischemic stroke. *Journal of Neurology* **2017**, *264*, doi:10.1007/s00415-016-8366-y.
57. Protto, S.; Pienimäki, J.-P.; Seppänen, J.; Numminen, H.; Sillanpää, N. Low Cerebral Blood Volume Identifies Poor Outcome in Stent Retriever Thrombectomy. *CardioVascular and Interventional Radiology* **2017**, *40*, 502-509, doi:10.1007/s00270-016-1532-x.
58. Zaidat, O.O.; Castonguay, A.C.; Linfante, I.; Gupta, R.; Martin, C.O.; Holloway, W.E.; Mueller-Kronast, N.; English, J.D.; Dabus, G.; Malisch, T.W.; et al. First Pass Effect: A New Measure for Stroke Thrombectomy Devices. *Stroke* **2018**, *49*, 660-666, doi:10.1161/strokeaha.117.020315.
59. Brinjikji, W.; Robert, M.S.; Murad, M.H.; David, F.; Vitor, M.P.; Mayank, G.; David, F.K. Impact of balloon guide catheter on technical and clinical outcomes: a systematic review and meta-analysis. *Journal of NeuroInterventional Surgery* **2018**, *10*, 335, doi:10.1136/neurintsurg-2017-013179.
60. van Horn, N.; Kniep, H.; Leischner, H.; McDonough, R.; Deb-Chatterji, M.; Broocks, G.; Thomalla, G.; Brekenfeld, C.; Fiehler, J.; Hanning, U.; et al. Predictors of poor clinical outcome despite complete reperfusion in acute ischemic stroke patients. *Journal of NeuroInterventional Surgery* **2021**, *13*, 14, doi:10.1136/neurintsurg-2020-015889.
61. Uniken Venema, S.M.; Wolff, L.; van den Berg, S.A.; Reinink, H.; Luijten, S.P.R.; Lingsma, H.F.; Marquering, H.A.; Boers, A.M.M.; Bot, J.; Hammer, S.; et al. Time Since Stroke Onset, Quantitative Collateral Score, and Functional Outcome After Endovascular Treatment for Acute Ischemic Stroke. *Neurology* **2022**, *99*, e1609-e1618, doi:10.1212/wnl.0000000000200968.
62. Shi, Z.-S.; Loh, Y.; Walker, G.; Duckwiler, G.R. Clinical outcomes in middle cerebral artery trunk occlusions versus secondary division occlusions after mechanical thrombectomy: pooled analysis of the Mechanical Embolus Removal in Cerebral Ischemia (MERCI) and Multi MERCI trials. *Stroke* **2010**, *41*, 953-960.
63. Binder, N.F.; El Amki, M.; Glück, C.; Middleham, W.; Reuss, A.M.; Bertolo, A.; Thurner, P.; Deffieux, T.; Lambride, C.; Epp, R.; et al. Leptomeningeal collaterals regulate reperfusion in ischemic stroke and rescue the brain from futile recanalization. *Neuron* **2024**, *112*, 1456-1472.e1456, doi:10.1016/j.neuron.2024.01.031.

64. Ravindran, A.V.; Killingsworth, M.C.; Bhaskar, S. Cerebral collaterals in acute ischaemia: Implications for acute ischaemic stroke patients receiving reperfusion therapy. *European Journal of Neuroscience* **2021**, *53*, 1238-1261, doi:https://doi.org/10.1111/ejn.14955.
65. Baek, J.H.; Kim, B.M.; Heo, J.H.; Nam, H.S.; Kim, Y.D.; Park, H.; Bang, O.Y.; Yoo, J.; Kim, D.J.; Jeon, P.; et al. Number of Stent Retriever Passes Associated With Futile Recanalization in Acute Stroke. *Stroke* **2018**, *49*, 2088-2095, doi:10.1161/strokeaha.118.021320.
66. Kitano, T.; Todo, K.; Yoshimura, S.; Uchida, K.; Yamagami, H.; Sakai, N.; Sakaguchi, M.; Nakamura, H.; Kishima, H.; Mochizuki, H.; et al. Futile complete recanalization: patients characteristics and its time course. *Scientific Reports* **2020**, *10*, 4973, doi:10.1038/s41598-020-61748-y.
67. Ni, H.; Liu, X.; Hang, Y.; Jia, Z.; Cao, Y.; Shi, H.; Liu, S.; Zhao, L. Predictors of futile recanalization in patients with acute ischemic stroke undergoing mechanical thrombectomy in late time windows. *Frontiers in Neurology* **2022**, *13*, 958236, doi:10.3389/fneur.2022.958236.
68. Jahan, R.; Saver, J.L.; Schwamm, L.H.; Fonarow, G.C.; Liang, L.; Matsouaka, R.A.; Xian, Y.; Holmes, D.N.; Peterson, E.D.; Yavagal, D.; et al. Association Between Time to Treatment With Endovascular Reperfusion Therapy and Outcomes in Patients With Acute Ischemic Stroke Treated in Clinical Practice. *JAMA* **2019**, *322*, 252-263, doi:10.1001/jama.2019.8286.
69. Meinel, T.R.; Kaesmacher, J.; Chaloulos-Iakovidis, P.; Panos, L.; Mordasini, P.; Mosimann, P.J.; Michel, P.; Hajdu, S.; Ribo, M.; Requena, M.; et al. Mechanical thrombectomy for basilar artery occlusion: efficacy, outcomes, and futile recanalization in comparison with the anterior circulation. *Journal of NeuroInterventional Surgery* **2019**, *11*, 1174-1180, doi:10.1136/neurintsurg-2018-014516.
70. Wang, D.; Shu, H.; Meng, Y.; Zhang, H.; Wang, H.; He, S. Factors Promoting Futile Recanalization After Stent Retriever Thrombectomy for Stroke Affecting the Anterior Circulation: A Retrospective Analysis. *World Neurosurg* **2020**, *133*, e576-e582, doi:10.1016/j.wneu.2019.09.098.
71. Kharouba, R.; Gavriliuc, P.; Yaghmour, N.E.; Gomori, J.M.; Cohen, J.E.; Leker, R.R. Number of stentriever passes and outcome after thrombectomy in stroke. *Journal of Neuroradiology* **2019**, *46*, 327-330, doi:10.1016/j.neurad.2019.03.014.
72. Garcia-Tornel Garcia-Camba, A.; Requena, M.; Rubiera, M.; Muchada, M.; Pagola, J.; Rodriguez-Luna, D.; Deck, M.; Juega, J.; Rodríguez-Villatoro, N.; Boned Riera, S.; et al. When to Stop: Detrimental Effect of Device Passes in Acute Ischemic Stroke Secondary to Large Vessel Occlusion. *Stroke* **2019**, *50*, doi:10.1161/STROKEAHA.119.025088.
73. Abbasi, M.; Liu, Y.; Fitzgerald, S.; Mereuta, O.M.; Arturo Larco, J.L.; Rizvi, A.; Kadirvel, R.; Savastano, L.; Brinjikji, W.; Kallmes, D.F. Systematic review and meta-analysis of current rates of first pass effect by thrombectomy technique and associations with clinical outcomes. *Journal of NeuroInterventional Surgery* **2021**, *13*, 212-216, doi:10.1136/neurintsurg-2020-016869.
74. Flottmann, F.; Leischner, H.; Broocks, G.; Nawabi, J.; Bernhardt, M.; Faizy, T.D.; Deb-Chatterji, M.; Thomalla, G.; Fiehler, J.; Brekenfeld, C. Recanalization Rate per Retrieval Attempt in Mechanical Thrombectomy for Acute Ischemic Stroke. *Stroke* **2018**, *49*, 2523-2525, doi:10.1161/strokeaha.118.022737.
75. Wang, Z.; Fan, L. Does stress hyperglycemia in diabetic and non-diabetic acute ischemic stroke patients predict unfavorable outcomes following endovascular treatment? *Neurological Sciences* **2023**, doi:10.1007/s10072-023-06625-y.
76. Filioglo, A.; Cohen, J.E.; Honig, A.; Simaan, N.; Gomori, J.M.; Leker, R.R. More than five stentriever passes: real benefit or futile recanalization? *Neuroradiology* **2020**, *62*, 1335-1340, doi:10.1007/s00234-020-02469-x.
77. Ribo, M.; Molina, C.A.; Cobo, E.; Cerdà, N.; Tomasello, A.; Quesada, H.; De Miquel, M.A.; Millan, M.; Castaño, C.; Urra, X.; et al. Association Between Time to Reperfusion and Outcome Is Primarily Driven by the Time From Imaging to Reperfusion. *Stroke* **2016**, *47*, 999-1004, doi:10.1161/STROKEAHA.115.011721.
78. Zhang, M.; Xing, P.; Tang, J.; Shi, L.; Yang, P.; Zhang, Y.; Zhang, L.; Peng, Y.; Liu, S.; Zhang, L.; et al. Predictors and outcome of early neurological deterioration after endovascular thrombectomy: a secondary analysis of the DIRECT-MT trial. *Journal of NeuroInterventional Surgery* **2022**, neurintsurg-2022-018976, doi:10.1136/neurintsurg-2022-018976.
79. Lee, S.-H.; Kim, B.J.; Han, M.-K.; Park, T.H.; Lee, K.B.; Lee, B.-C.; Yu, K.-H.; Oh, M.S.; Cha, J.K.; Kim, D.-H. Futile reperfusion and predicted therapeutic benefits after successful endovascular treatment according to initial stroke severity. *BMC Neurology* **2019**, *19*, 1-9.

80. Dong, A.; Maier, B.; Guillon, B.; Preterre, C.; De Gaalon, S.; Gory, B.; Richard, S.; Kaminsky, A.L.; Tracol, C.; Eugene, F.; et al. TICI-RANKIN mismatch: Poor clinical outcome despite complete endovascular reperfusion in the ETIS Registry. *Revue Neurologique* **2023**, *179*, 230-237, doi:10.1016/j.neurol.2022.10.003.
81. Heitkamp, C.; Winkelmeier, L.; Heit, J.J.; Albers, G.W.; Lansberg, M.G.; Wintermark, M.; Broocks, G.; van Horn, N.; Kniep, H.C.; Sporns, P.B.; et al. Unfavorable cerebral venous outflow is associated with futile recanalization in acute ischemic stroke patients. *European Journal of Neurology* **2023**, *n/a*, doi:10.1111/ene.15898.
82. Mohammaden, M.H.; Stapleton, C.J.; Brunozzi, D.; Hussein, A.E.; Khedr, E.M.; Atwal, G.; Alaraj, A. Predictors of Poor Outcome Despite Successful Mechanical Thrombectomy of Anterior Circulation Large Vessel Occlusions Within 6 h of Symptom Onset. *Frontiers in Neurology* **2020**, *11*, 907, doi:10.3389/fneur.2020.00907.
83. Spronk, E.; Sykes, G.; Falcione, S.; Munsterman, D.; Joy, T.; Kamtchum-Tatuene, J.; Jickling, G.C. Hemorrhagic Transformation in Ischemic Stroke and the Role of Inflammation. *Frontiers in Neurology* **2021**, *12*, doi:10.3389/fneur.2021.661955.
84. Vatan, M.B.; Acar, B.A.; Acar, T.; Aras, Y.G. The CHA2DS2-VASc risk score predicts futile recanalization after endovascular treatment in patients with acute ischemic stroke. *Neurology Asia* **2023**, *28*, doi:10.54029/2023kep.
85. Boisseau, W.; Jean-Philippe, D.; Robert, F.; Maeva, K.; Kevin, Z.; Candice, S.; Guillaume, T.; Malek Ben, M.; Benjamin, M.; Daniele, B.; et al. Neutrophil count predicts poor outcome despite recanalization after endovascular therapy. *Neurology* **2019**, *93*, e467, doi:10.1212/WNL.0000000000007859.
86. Zhou, T.; Tingyu, Y.; Tianxiao, L.; Liangfu, Z.; Yucheng, L.; Zhaoshuo, L.; Meiyun, W.; Qiang, L.; Yingkun, H.; Pengfei, Y.; et al. Predictors of futile recanalization in patients undergoing endovascular treatment in the DIRECT-MT trial. *Journal of NeuroInterventional Surgery* **2022**, *14*, 752, doi:10.1136/neurintsurg-2021-017765.
87. Mechtouff, L.; Bochaton, T.; Paccalet, A.; Da Silva, C.C.; Buisson, M.; Amaz, C.; Derex, L.; Ong, E.; Berthezene, Y.; Eker, O.F.; et al. Association of Interleukin-6 Levels and Futile Reperfusion After Mechanical Thrombectomy. *Neurology* **2021**, *96*, e752-e757, doi:10.1212/wnl.0000000000011268.
88. Tajima, Y.; Hayasaka, M.; Ebihara, K.; Kubota, M.; Suda, S. Predictors of poor outcome after successful mechanical thrombectomy in patients with acute anterior circulation stroke. *Journal of Clinical Interventional Radiology* **2017**, *1*, 139-143.
89. Shi, Z.S.; Liebeskind, D.S.; Xiang, B.; Ge, S.G.; Feng, L.; Albers, G.W.; Budzik, R.; Devlin, T.; Gupta, R.; Jansen, O.; et al. Predictors of functional dependence despite successful revascularization in large-vessel occlusion strokes. *Stroke* **2014**, *45*, 1977-1984, doi:10.1161/strokeaha.114.005603.
90. Baskar, P.S.; Chowdhury, S.Z.; Bhaskar, S.M.M. In-hospital systems interventions in acute stroke reperfusion therapy: a meta-analysis. *Acta Neurologica Scandinavica* **2021**, *144*, 418-432, doi:https://doi.org/10.1111/ane.13476.
91. Phuong, N.V.; Cong Thanh, N.; Keserci, B.; Sang, N.V.; Minh Duc, N. Mechanical thrombectomy treatment of basilar artery occlusion within 24 hours of symptom onset: A Single-Center Experience. *Clin Ter* **2022**, *173*, 400-406, doi:10.7417/ct.2022.2454.
92. Aguirre, C.; Trillo, S.; Ramos, C.; Zapata-Wainberg, G.; Sanz-García, A.; Ximénez-Carrillo, Á.; Barbosa, A.; Caniego, J.L.; Vivancos, J. Predictive value of ischemia location on multimodal CT in thrombectomy-treated patients. *The Neuroradiology Journal* **2022**, 19714009221128658, doi:10.1177/19714009221128658.
93. de Havenon, A.; Elhorany, M.; Boulouis, G.; Naggara, O.; Darcourt, J.; Clarençon, F.; Richard, S.; Marnat, G.; Bourcier, R.; Sibon, I.; et al. Thrombectomy in basilar artery occlusions: impact of number of passes and futile reperfusion. *Journal of NeuroInterventional Surgery* **2022**, neurintsurg-2022-018715, doi:10.1136/neurintsurg-2022-018715.
94. Seker, F.; Qureshi, M.M.; Möhlenbruch, M.A.; Nogueira, R.G.; Abdalkader, M.; Ribo, M.; Caparros, F.; Haussen, D.C.; Mohammaden, M.H.; Sheth, S.A.; et al. Reperfusion Without Functional Independence in Late Presentation of Stroke With Large Vessel Occlusion. *Stroke* **2022**, *53*, 3594-3604, doi:10.1161/strokeaha.122.039476.
