# Supplementary material for: Introducing the Futile Recanalization Prediction Score (FRPS): A Novel Approach to Predict and Mitigate Ineffective Recanalization after Endovascular Treatment of Acute Ischemic Stroke
Source: Neurol Int. 2024 May 30;16(3):605–19. doi: 10.3390/neurolint16030045 (PMC11206671; doi:10.3390/neurolint16030045)
Supplement: Supplementary file 1 [file neurolint-16-00045-s001.zip › Supplemental Information_2.pdf]

Supplemental Information 2

Introducing the Futile Recanalization Prediction Score (FRPS): A Novel Approach to Predict and Mitigate Ineffective Recanalization after Endovascular Treatment of Acute Ischemic Stroke

Supplemental Information 2: Python Codes

1. Random Forest Regression Modelling and Validation

```
import pandas as pd
import numpy as np
from sklearn.ensemble import RandomForestRegressor

from sklearn.model_selection import cross_val_score

# Generate synthetic data with 5000 data points
np.random.seed(0)
n_samples = 5000

# Generate random values for predictor variables
age = np.random.randint(40, 90, size=n_samples)
nihss_score = np.random.randint(0, 25, size=n_samples)
sex = np.random.randint(0, 2, size=n_samples)  # 0: Male, 1: Female
af = np.random.randint(0, 2, size=n_samples)  # Atrial Fibrillation
htn = np.random.randint(0, 2, size=n_samples)  # Hypertension
dm = np.random.randint(0, 2, size=n_samples)  # Diabetes
hyperlipidemia = np.random.randint(0, 2, size=n_samples)
cognitive_impairment = np.random.randint(0, 2, size=n_samples)
pre_stroke_mrs = np.random.randint(0, 6, size=n_samples)  # Modified Rankin Scale
score
sbp = np.random.randint(100, 200, size=n_samples)  # Systolic Blood Pressure
onset_to_puncture_time = np.random.randint(0, 360, size=n_samples)  # Onset to
Puncture Time (minutes)
sich = np.random.randint(0, 2, size=n_samples)  # Symptomatic Intracranial
Hemorrhage
```

Supplemental Information 2

```
# Generate FRPS scores based on a linear combination of predictor variables
frps_score = 0.49 * age + 1.39 * af + 1.15 * htn + 1.65 * dm + 1.71 * hyperlipidemia + \
             1.40 * sex + 1.30 * cognitive_impairment + 0.66 * (1 - sex) + 0.33 * (1 - sex) + \
             0.75 * np.random.randint(0, 2, size=n_samples) + 0.31 * sbp + 0.20 * onset_to_puncture_time + \
             0.75 * (nihss_score > 14) - 0.37 * pre_stroke_mrs + 0.75 * (nihss_score > 14) * sich

# Create DataFrame
df = pd.DataFrame({
    'Age': age,
    'Sex': sex,
    'AF': af,
    'HTN': htn,
    'DM': dm,
    'Hyperlipidemia': hyperlipidemia,
    'Cognitive Impairment': cognitive_impairment,
    'Pre-Stroke MRS': pre_stroke_mrs,
    'SBP': sbp,
    'Onset to Puncture Time': onset_to_puncture_time,
    'sICH': sich,
    'NIHSS Score': nihss_score,
    'FRPS Score': frps_score
})

# Select predictor variables
selected_predictors = ['Age', 'Sex', 'AF', 'HTN', 'DM', 'Hyperlipidemia', 'Cognitive Impairment',
                       'Pre-Stroke MRS', 'SBP', 'Onset to Puncture Time', 'sICH', 'NIHSS Score']

# Prepare the predictor matrix (X) and target variable (y)
X = df[selected_predictors]
y = df['FRPS Score']
```

Supplemental Information 2

```
# Initialize the Random Forest regression model
rf_model = RandomForestRegressor()

# Perform cross-validation to evaluate the model
cv_scores = cross_val_score(rf_model, X, y, cv=5, scoring='r2')

# Compute the mean R-squared value
mean_r2 = np.mean(cv_scores)

print("Mean R-squared value for the Random Forest model:", mean_r2)

# # # # # # # # # # # # # # # # # # # # # # # # # # # # # # # # # # # # # # # # # # # #
# # #
# # # # # # # # # #  END OF THE CODE # # # # # # # #
```

Output

Output: Mean R-squared value for the Random Forest model: 0.991628575570196

2.

Feature Sorting Based on Importance

```
# Fit the Random Forest model
rf_model.fit(X, y)

# Get feature importances
feature_importances = rf_model.feature_importances_

# Create a DataFrame to display the feature importances
feature_importance_df = pd.DataFrame({'Feature': selected_predictors, 'Importance':
feature_importances})
```

Supplemental Information 2

```
feature_importance_df = feature_importance_df.sort_values(by='Importance',
ascending=False)

# Display feature importances
print(feature_importance_df)

###########################################
###
########## END OF THE CODE ########
#Output
```

```
                Feature
Importance
9               Onset to Puncture Time
0.768544
8               SBP
0.139711
0               Age
0.086357
11              NIHSS Score
0.001538
7               Pre-Stroke MRS
```

3. FRPS Score Calculation and Risk Severity Classification

```
import numpy as np

# Function to calculate FRPS score
def calculate_frps_score(age, onset_to_puncture_time, sbp, nihss_score,
pre_stroke_mrs, dm, hyperlipidemia, af, cognitive_impairment, htn, sex, sich):
    frps = (0.769 * onset_to_puncture_time) + \
           (0.140 * sbp) + \
           (0.086 * age) + \
           (0.002 * nihss_score) + \
           (0.001 * pre_stroke_mrs) + \
```

```
        (0.001 * dm) + \
        (0.0005 * hyperlipidemia) + \
        (0.0005 * af) + \
        (0.0004 * cognitive_impairment) + \
        (0.0004 * htn) + \
        (0.0003 * sex) + \
        (0.0003 * sich)
    return frps

# Simulate FRPS scores for 1000 hypothetical patients
num_patients = 1000
np.random.seed(42)  # For reproducibility

# Simulated patient characteristics
ages = np.random.randint(40, 90, num_patients)
onset_to_puncture_times = np.random.uniform(0, 120, num_patients)
sbps = np.random.uniform(90, 200, num_patients)
nihss_scores = np.random.randint(0, 42, num_patients)
pre_stroke_mrss = np.random.randint(0, 6, num_patients)
dms = np.random.choice([0, 1], size=num_patients)
hyperlipidemias = np.random.choice([0, 1], size=num_patients)
afs = np.random.choice([0, 1], size=num_patients)
cognitive_impairments = np.random.choice([0, 1], size=num_patients)
htns = np.random.choice([0, 1], size=num_patients)
sexes = np.random.choice([0, 1], size=num_patients)
sichs = np.random.choice([0, 1], size=num_patients)

# Calculate FRPS scores
frps_scores = []
for i in range(num_patients):
    frps = calculate_frps_score(ages[i], onset_to_puncture_times[i], sbps[i],
nihss_scores[i], pre_stroke_mrss[i],
                                dms[i], hyperlipidemias[i], afs[i], cognitive_impairments[i], htns[i],
sexes[i], sichs[i])
    frps_scores.append(frps)

# Define severity ranges based on percentiles of FRPS scores
```

```
mild_range = np.percentile(frps_scores, [20, 40])
moderate_range = np.percentile(frps_scores, [40, 60])
severe_range = np.percentile(frps_scores, [60, 80])

print("Mild Range:", (mild_range[0], mild_range[1]))
print("Moderate Range:", (moderate_range[0], moderate_range[1]))
print("Severe Range:", (severe_range[0], severe_range[1]))

###########################################
###
########## END OF THE CODE ########
```

#Output

```
Mild Range: (46.02824665144297, 65.7444382505024)
Moderate Range: (65.7444382505024, 80.94397005586168)
Severe Range: (80.94397005586168, 100.20404510722125)
```
